# Supplementary material for: Clinical and cost-effectiveness of a personalised guided consultation versus usual physiotherapy care in people presenting with shoulder pain: a protocol for the PANDA-S cluster randomised controlled trial and process evaluation
Source: BMJ Open. 2025 May 6;15(5):e100501. doi: 10.1136/bmjopen-2025-100501 (PMC12056648; doi:10.1136/bmjopen-2025-100501)
Supplement: online supplemental file 1 [file bmjopen-15-5-s001.docx]

| **Consent Form** | | |
| --- | --- | --- |
| Please ensure you have read the enclosed **Participant Information Leaflet** before you complete the consent form. This leaflet contains key information about the study and what taking part in the PANDA-S II Study involves. | | |
|  | Please cross one box | |
|  | Yes | No |
| - I confirm that I have read and understood the Participant Information Leaflet (Version 2.0 dated 21 Apr 2023) and am willing to take part in the PANDA-S II Study ……………… |  |  |
| - I am willing to receive further information about related, optional aspects of the study (audio-recording of the consultation, interview with one of the research team) ….. |  |  |
| - I am willing for my GP to be notified about my participation in the study …………………………………………………….   (*If you consent to your GP being notified about your participation in the study, please complete your GP details on the next page) |  |  |
| - I understand that I can withdraw from the study at any time without giving a reason, and that this will not affect the care I receive in any way ……………………………………………. |  |  |
| - I understand that relevant sections of my medical notes and data collected during the study may possibly be looked at by individuals from regulatory authorities or from the NHS Trust where it is relevant to my taking part in this research. I give my permission for these individuals to have access to my records …………………………………………………………… |  |  |


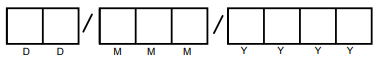


Signature: __________________________________

***[Please enter today’s date e.g. 06 / MAR / 2023]***

Print name: _______________________________________________________________

This consent form will be stored securely at Keele Clinical Trials Unit (CTU).

Please contact 01782 732950 if you would like a copy
