# Supplementary material for: Clinical and cost-effectiveness of a personalised guided consultation versus usual physiotherapy care in people presenting with shoulder pain: a protocol for the PANDA-S cluster randomised controlled trial and process evaluation
Source: BMJ Open. 2025 May 6;15(5):e100501. doi: 10.1136/bmjopen-2025-100501 (PMC12056648; doi:10.1136/bmjopen-2025-100501)
Supplement: Supplementary file 2 [file bmjopen-2025-100501.reviewer_comments.pdf]

## PEER REVIEW HISTORY

BMJ Open publishes all reviews undertaken for accepted manuscripts. Reviewers are asked to complete a checklist review form and are provided with free text boxes to elaborate on their assessment. These free text comments are reproduced below.

### ARTICLE DETAILS

**Title (Provisional)**

CLINICAL AND COST-EFFECTIVENESS OF A PERSONALISED GUIDED CONSULTATION VERSUS USUAL PHYSIOTHERAPY CARE IN PEOPLE PRESENTING WITH SHOULDER PAIN: A PROTOCOL FOR THE PANDA-S CLUSTER RANDOMISED CONTROLLED TRIAL AND PROCESS EVALUATION

**Authors**

Harrisson, Sarah; Myers, Helen; Wynne-Jones, Gwenllian; Bajpai, Ram; Bratt, Claire; Burton, Claire; Harrison, Rosie; Jowett, Sue; Lawton, Sarah Ann; Saunders, Benjamin; Beard, David; Bucknall, Milica; Chester, Rachel; Heneghan, Carl; Huckfield, Lucy; Lewis, Martyn; Mallen, Christian; Pincus, Tamar; Rees, J L; Roddy, Edward; van der Windt, Danielle

---

This article was not externally reviewed at BMJ Open. Protocols that have been independently assessed prior to submission to BMJ Open are usually fast-tracked to publication on the grounds that further substantial changes will not be possible. This independent assessment will usually be external, independent review for both a substantial grant award from a non-commercial or government funder and ethics approval.
